# Alternative routes to intravenous tranexamic acid for postpartum hemorrhage: A systematic search and narrative review

**DOI:** 10.1002/ijgo.14201

**Published:** 2022-06-28

**Authors:** Haleema Shakur‐Still, Stanislas Grassin‐Delyle, Kopalasuntharam Muhunthan, Homa K. Ahmadzia, David Faraoni, Monica Arribas, Ian Roberts

**Affiliations:** ^1^ Clinical Trials Unit London School of Hygiene and Tropical Medicine London UK; ^2^ Département des maladies des voies respiratoires Hôpital Foch Suresnes France; ^3^ Infection et inflammation, Département de Biotechnologie de la Santé, UVSQ, INSERM Université Paris‐Saclay Montigny le Bretonneux France; ^4^ Department of Obstetrics and Gynecology University of Jaffna Jaffna Sri Lanka; ^5^ Department of Obstetrics and Gynecology, Division of Maternal‐Fetal Medicine George Washington University Washington District of Columbia USA; ^6^ Department of Anesthesiology, Perioperative and Pain Medicine, Arthur S. Keats Division of Pediatric Cardiovascular Anesthesia, Texas Children’s Hospital Baylor College of Medicine Houston Texas USA

**Keywords:** administration routes, antifibrinolytic pharmacokinetics, pharmacodynamics, postpartum hemorrhage, tranexamic acid

## Abstract

**Objective:**

To review available data on tranexamic acid (TXA) plasma concentration needed to inhibit fibrinolysis and the time to achieve this concentration when giving TXA by different routes in humans. To identify ongoing trials assessing alternatives to intravenous TXA administration.

**Methods:**

We updated two previous systematic reviews by searching MEDLINE, EMBASE, OviSP, and ISI Web of Science from database inception to July 2021. We also searched the WHO International Clinical Trials Registry Platform for ongoing trials to July 2021. Titles and abstracts were screened for relevant trials. Two reviewers independently reviewed and agreed the trials to be included.

**Results:**

Plasma TXA concentrations over 10 mg/L provide near maximal inhibition of fibrinolysis, with concentrations over 5 mg/L providing partial inhibition. Oral TXA tablets take about 1 h to reach a plasma concentration of 5 mg/L in postpartum women. Studies in healthy volunteers and shocked trauma patients show that intramuscular TXA achieves a plasma level of over 10 mg/L within 15 min. One trial is ongoing to determine the pharmacokinetics of intramuscular and oral solution TXA in pregnant women.

**Conclusion:**

Intramuscular TXA in healthy volunteers and shocked trauma patients reaches therapeutic concentration rapidly. Oral TXA tablets take too long to reach the minimum therapeutic concentration in postpartum women.

## Synopsis

Intravenous tranexamic acid (TXA) reduces death due to bleeding if given as soon as possible after birth and no later than 3 hours. TXA orally takes about 1 hour to reach minimum therapeutic concentration in postpartum women. Intramuscular TXA achieves therapeutic concentration within 5 minutes in healthy volunteers and in shocked trauma patients.

## 1 INTRODUCTION

Postpartum hemorrhage (PPH) affects about 14 million women every year and about 70 000 will die from exsanguination.^1^ PPH is one of the main causes of maternal mortality in all countries, although the absolute risk of death from PPH is much higher in low‐resourced settings.^2^ All women bleed after childbirth and the diagnosis of PPH is usually made when bleeding is greater than expected or results in signs and symptoms of shock. The definition of PPH proposed by a World Health Organization (WHO) working group in 1989 is an estimated blood loss of ≥500 ml after vaginal birth or ≥ 1000 ml after cesarean birth.^3^ Using blood loss criteria to define PPH is problematic because bleeding may not be visible, such as when it is intra‐abdominal, or when blood in collection devices is mixed with amniotic fluid, or when bleeding occurs after closure of the abdomen in a cesarean birth. The same threshold is proposed for all women. Despite recognizing the need for an alternative definition of PPH in anemic women, no criteria were proposed.^3^ Although the WHO PPH definition includes “any blood loss sufficient to compromise haemodynamic stability”,^3^ how this should be assessed is not explained, especially for women with common medical conditions including anemia, hypertension, or eclampsia.

Early recognition and appropriate intervention are critical to prevent death and serious morbidities. In high‐income countries, most women give birth in hospital or have access to ambulance transport and doctors or paramedics so have rapid access to PPH treatments. However, in low‐ and middle‐income countries, a large number of women give birth at home, often with poor access to transport; therefore, getting to hospital can take hours and some women exsanguinate before arrival.^4^ In hospitals, although healthcare professionals are present, many do not have the skills needed or are not allowed to give PPH treatments requiring intravenous (IV) administration.

Tranexamic acid (TXA) is an antifibrinolytic drug that works by inhibiting clot lysis and stabilizes clot formation by blocking the fibrin‐binding sites on plasminogen and preventing its attachment to a newly developing clot. Specifically, TXA is a synthetic analogue of the amino acid lysine and primarily inhibits plasminogen. Less plasminogen ultimately means less plasmin, which is the active enzyme that breaks down fibrin.^5^ TXA is used to prevent bleeding complications and reduce mortality with minimal adverse effects in a variety of clinical conditions.

Prophylactic administration of TXA in surgical procedures reduces the probability of receiving a blood transfusion by one‐third (RR 0.62; 95% CI, 0.58–0.65) and reduces blood loss by a similar amount (pooled ratio 0.66; 95% CI 0.65–0.67).^6^, ^7^

When used to treat acute traumatic hemorrhage, the CRASH‐2 trial showed a one‐third reduction in bleeding mortality when TXA was given within 3 h of injury. Also, the CRASH‐3 trial showed a significant reduction in head injury‐related deaths when TXA was administered within 3 h of injury to patients with mild‐to‐moderate traumatic brain injury.^8^, ^9^, ^10^ When used to treat heavy menstrual bleeding, evidence suggests there is a 40%–50% reduction in the amount of menstrual blood lost per menstrual cycle for participants taking TXA.^11^

Although concerns are sometimes expressed about the risk of thrombotic adverse events associated with TXA use, a recent systematic review and meta‐analysis of 216 randomized trials including 125 550 patients found no increased risk of any thromboembolic events in patients who received TXA compared with placebo.^12^

### 1.1 Intravenous tranexamic acid for PPH

The WOMAN trial was a randomized placebo‐controlled trial of tranexamic acid to treat PPH. Women with primary PPH after vaginal or cesarean delivery were randomly allocated to receive 1 g TXA or matching placebo by IV injection. If bleeding continued after 30 min, they received a second dose of 1 g TXA IV or placebo. The trial recruited 20 060 women with PPH and contributed 99% of the data for the Cochrane systematic review of TXA for PPH, which included 20 172 women from two trials.^13^, ^14^ The review showed that IV TXA reduces the risk of maternal death due to bleeding (RR 0.81; 95% CI, 0.65–1.00). The effect was more evident in women given treatment between 1 and 3 h after giving birth with no apparent reduction when given after 3 h (<1 h = RR 0.80; 95% CI, 0.55–1.16; 1–3 h = RR 0.60; 95% CI, 0.41–0.88; >3 h = RR 1.07; 95% 0.76–1.51; test for subgroup differences: *χ* ^2^ = 4.90, df = 2 [*P* = 0.09], *I* ^2^ = 59.2%). There was no heterogeneity in the effect by mode of birth (test for subgroup differences: *χ* ^2^ = 0.01, df = 1 [*P* = 0.91], *I* ^2^ = 0%). TXA also reduced the need for laparotomy for bleeding (RR 0.64; 95% CI, 0.49–0.85). There was no increase in any maternal vascular occlusive events, although results were imprecise (RR 0.88; 95% CI, 0.54–1.43).^14^ TXA is known to pass into breast milk in very low concentrations, approximately one hundredth of the concentration in the maternal blood.^15^, ^16^ Vascular occlusive events in neonates were assessed in the WOMAN trial but no events were observed.^13^

In 2017, WHO recommended TXA for the treatment of PPH and stated that it “strongly recommends early use of IV tranexamic acid (within 3 h of birth) in addition to standard care for women with clinically‐diagnosed PPH following vaginal birth or caesarean section. Tranexamic acid should be used in all cases of PPH, regardless of whether the bleeding is thought to be due to genital tract trauma or other causes, including uterine atony”.^17^ The WHO recommendation emphasized a 3‐h threshold (following birth), after which tranexamic acid should not be given.^17^ This was based on meta‐analysis of individual patient‐level data from 40 138 bleeding patients from the WOMAN and CRASH‐2 trials, which showed that deaths from PPH peak at 2–3 h after childbirth and every 15 min of treatment delay with IV TXA appears to decrease the benefit by about 10%, with no benefit after 3 h.^18^

WHO also recommended that regardless of the level of health system resources, TXA should be recognized as a lifesaving intervention and be made readily available for the management of PPH in settings where emergency obstetric care is provided.^17^

The aim of the present narrative review was to investigate the TXA plasma concentration needed to inhibit fibrinolysis and the plasma levels achieved when TXA is administered using different routes to identify alternatives to IV administration. We also identify ongoing trials that aim to provide information on alternatives to IV routes of TXA administration.

## 2 MATERIALS AND METHODS

To determine the plasma TXA level needed to inhibit fibrinolysis we updated a previously published systematic review of in vitro and in vivo pharmacodynamics studies. This review searched MEDLINE, EMBASE, OviSP, and ISI Web of Science from database inception to November 2017 for all in vitro or in vivo studies that reported the relationship between the TXA concentration in blood or plasma.^19^ We also updated the review by Kane et al.^20^ to model the plasma levels achieved when TXA is administered using different routes. Kane et al.^20^ used the same search criteria as per the previous search but extended it to include studies to June 2018. For the purposes of the present review, we updated the searches of these earlier reviews from July 2018 to July 2021 (see Data S1 for search strategy). We also searched the WHO International Clinical Trials Registry Platform for ongoing trials. Titles and abstracts were screened for relevant trials. Two reviewers (HSS and IR) independently reviewed and agreed the trials to be included in this narrative review.

## 3 ALTERNATIVE ROUTES TO IV TXA AND THEIR EFFECTIVENESS

One of the access barriers to TXA treatment is the need for an IV injection. Health workers able to give IV drugs may be unavailable at home births in rural areas and in low‐level healthcare facilities, and even when they are, securing IV access can be difficult in shocked patients with collapsed veins. TXA is also available in tablet form for oral administration. Whereas IV administration bypasses the absorption step and is 100% bioavailable immediately, oral bioavailability is limited by intestinal permeability and was predicted to vary from 33%–34%.^20^ Use of the IV solution for intramuscular (IM), subcutaneous, and oral administration might achieve therapeutic blood levels more rapidly.

### 3.1 Minimum serum level of TXA

To understand the plasma level of TXA needed to attain therapeutic response, a systematic review of pharmacodynamics studies was conducted.^19^ Our updated search did not identify any new studies. In vivo and in vitro studies reporting both the concentration of TXA in blood or plasma and its effects on any reliable measure of fibrinolysis were reviewed. The systematic review suggests that TXA concentrations in the range of 10–15 mg/L result in substantial inhibition of fibrinolysis although concentrations between 5 mg/L and 10 mg/L were partly inhibitory. Therefore, TXA concentrations of 10–15 mg/L may be suitable targets for pharmacokinetic studies, although TXA concentrations above 5 mg/L may also be effective.^19^

### 3.2 Modeling of different routes of administration of TXA to achieve therapeutic serum levels

To assess if oral (tablet and solution), IM, or subcutaneous injections are viable treatment options, physiologically based pharmacokinetic modeling (PBPK) was used to evaluate if adequate TXA blood levels could be achieved when given via these different routes of administration. Single dose oral, IM, and subcutaneous simulations were performed and evaluated against a target plasma concentration of 15 mg/L.^20^

This study suggested that IM administration has the most potential of all the nonintravenous routes of administration for achieving therapeutic TXA levels. Plasma levels following an IM injection of 1 g TXA were predicted to exceed 15 mg/mL in <15 min. If oral solution of 4 g was administered, plasma TXA concentrations were predicted to exceed 15 mg/L within 15–30 min. The oral simulations used assumed rapid (0.1 h) gastric emptying of a solution formulation. The simulations for subcutaneous administration of TXA showed that it appears to be the route with the lowest potential for success. A dose of 2 g TXA was predicted to be comparable with or slower than those that could be achieved by the oral route, and therefore offered no advantages.^20^ If absorption was similarly rapid in pregnant women, this would suggest the IM and oral solution routes as potential alternatives to IV use.

### 3.3 TXA administered by different routes in healthy volunteers

**FIGURE 1:**
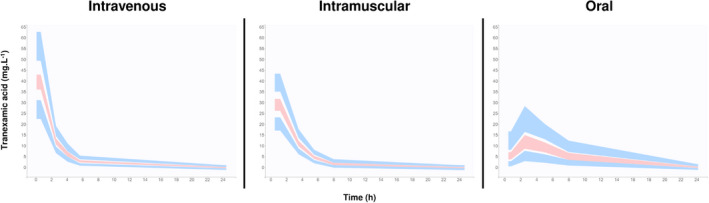
Tranexamic acid serum concentrations in healthy volunteers receiving 1 g intravenous, 1 g intramuscular, or 2 g oral tranexamic acid. The areas stand for the 95% confidence interval of the predictions of the 5th, 50th, and 95th percentiles of tranexamic acid concentrations. Source: Adapted from Grassin‐Delyle et al.^22^

To test the results of the PKPB modeling, a Phase 1 trial in healthy volunteers (Pharmaco‐TXA trial) was designed to explore the pharmacokinetics of TXA given by IV, IM, and oral solution.^22^ This randomized, open‐label, cross‐over trial recruited 15 adult healthy volunteers (11 nonpregnant women and 4 men) aged between 22 and 44 years. Volunteers were randomized to receive 1 g TXA IV, 1 g TXA IM (two x 5 ml intramuscular injections administered in the deltoid [9 volunteers] or vastus lateralis [6 volunteers]), or 2 g TXA oral solution. Ten blood samples were taken between 5 min and 24 h for measurement of serum TXA concentration, and a compartmental population pharmacokinetic model was constructed by nonlinear mixed effect modeling. The concentration vs time profiles for each administration route are shown in Figure 1. The time to reach 10 mg/L was 1, 3.5, and 66 min for the IV, IM, and oral routes, respectively. Whereas the oral solution route only allowed the concentration of 10 mg/L to be exceeded in 11 volunteers, with a median delay of 66 min explained by a lag‐time of 26 min before the absorption phase, the IM route allowed this threshold to be reached rapidly in all the volunteers. In pharmacokinetic terms, this is reflected in the excellent bioavailability observed for the IM route (100%) compared with the lower bioavailability of the oral route (47%). In addition, the IM route also results in a lower peak plasma level than the IV route, which could also improve the tolerance profile of the drug. There were no serious adverse effects and only moderate and short‐lived pain was observed after the IM injections.^22^

### 3.4 Pharmacokinetics and pharmacodynamics in pregnancy

During pregnancy, anatomic and physiologic changes occur in nearly all organ systems and it may not be reasonable to extrapolate data from healthy volunteers to pregnant women. The changes that occur with pregnancy can greatly influence pharmacologic principles of absorption, distribution, metabolism, and excretion.^21^, ^23^ Specifically, pregnant women have increased plasma volume by 30%–50%, increased renal plasma flow up to 50%, and decreased gastric motility. These changes, which are in response to the metabolic demands of the growing fetus and often secondary to the hormonal changes from placental release of progesterone, result in increased renal volumes of distribution and clearance from the beginning of pregnancy. Indeed, this renal elimination is achieved by the phenomena of glomerular filtration, active tubular secretion, and/or reabsorption. Glomerular filtration is the main mechanism for renal elimination. Glomerular filtration rate increases up to 50% during the first trimester of pregnancy, with this increase continuing in a more moderate way until the end of the pregnancy.^24^ With regard to the volume of distribution, an increase in total body water of approximately 3.5, 8.4, and 14.3 L is observed at 12, 25, and 40 weeks of pregnancy, respectively, related to the fetus and its appendages, as well as to an increase in intracellular, interstitial, and plasma water volume.^25^

TXA is a small hydrophilic molecule, not bound to plasma proteins and eliminated exclusively by the renal route.^26^ It is therefore likely that the exposure of pregnant women to TXA is reduced compared with a population of healthy volunteers, and specific pharmacokinetic studies should be conducted in this population.

Better estimates of the optimal TXA plasma concentration or range of concentrations required to fully inhibit fibrinolytic activation in this population is needed. In a recent study by Lechien et al.,^27^ the authors examined the TXA concentration required to inhibit tissue plasminogen activator (t‐PA) induced fibrinolysis in full‐term pregnant women using an in vitro model simulating a massive fibrinolytic activation on rotational thromboelastometry (ROTEM). Hyperfibrinolysis was induced by adding supraphysiologic concentration of t‐PA to blood samples obtained from 30 full‐term pregnant women and 10 healthy nonpregnant female volunteers. Increasing TXA concentrations (0–40 mg/L) were then spiked into the blood samples and inhibition of fibrinolysis was assessed using the lysis index at 30 min of the ROTEM measured on EXTEM and NATEM tests. Effective TXA concentrations required to achieve 95% inhibition of fibrinolysis (EC95) were extrapolated using nonlinear regression. EC95 were compared between groups using an extra sum‐of‐squares F test. EC95 in pregnant women was 14.7 mg/L (95% CI, 12.4–17.5 mg/L) on EXTEM and 11.2 mg/L (95% CI, 8.3–15.1 mg/L) on NATEM tests. The concentrations required were significantly higher than those obtained in the healthy volunteers: 8.7 mg/L (95% CI, 5.5–13.9 mg/L) and 6.8 mg/L (95% CI, 5.3–8.8 mg/L), respectively (both *P* < 0.001).^27^

### 3.5 Pharmacokinetics of oral TXA tablets in postpartum women

A single‐center study conducted in Sri Lanka included 12 healthy postpartum women and looked at the pharmacokinetics of 2 g of immediate‐release TXA tablets administered orally 1 h after vaginal delivery. Pharmacokinetic parameters were measured on plasma samples at baseline and then at 13 timepoints up to 12 h after administration. The mean maximum observed plasma concentration was 10.1 mg/L (range 8.6–12.2 mg/L). The mean time to maximum plasma concentration was 2.9 h (range 2.5–3.5 h). Mean time taken to reach the plasma concentration of 5 mg/L was 0.9 h.^28^ Although levels reached were within range to partially inhibit fibrinolysis, the time taken to reach these levels would lead to significant delay when treating PPH with TXA because with every 15 min of treatment delay the benefit of TXA decreases by about 10%, with no benefit after 3 h.^18^

### 3.6 TXA absorption in shock and hemorrhaging

PPH can lead to hypovolemic shock, which can lead to reduced blood flow to peripheral tissues. Skin and skeletal muscle are main targets for this response.^29^ This could reduce the absorption of TXA if given intramuscularly. To assess if this was the case, the pharmacokinetics of IM TXA was studied in bleeding trauma patients.^30^

An open‐label pharmacokinetic study that included 30 bleeding trauma patients (26 males, 4 females) was conducted. Patients received a loading dose of 1 g IV TXA and the second TXA dose was given intramuscularly as two 5 ml (0.5 g each) injections. The IM injections were given into noninjured muscles using the Z‐track method to reduce TXA leakage. Intramuscular TXA was well tolerated. Clinical signs of shock were seen in 18 patients. Of the 30 patients who received IM TXA, two had erythema, four had induration and subcutaneous nodules, and eight had bruising at the injection site. No patients had erythema, induration, or subcutaneous nodules beyond the day of injection. There was one adverse event of pyrexia 2 days after the injection and no serious adverse events were reported. Simulated concentration‐time profiles after a single IM TXA dose of 1 g show a TXA concentration of 5 mg/L would be achieved in about 4 min and a TXA concentration of 10 mg/L would be achieved in about 11 min.^30^

This trial showed that IM TXA is well tolerated with only mild and transient injection site reactions. Intramuscular TXA was rapidly absorbed, reaching therapeutic concentrations in less than 15 min. Blood lactate and signs of shock had no apparent impact on the rate of absorption.^30^

### 3.7 Ongoing trials

#### Pharmacokinetics and pharmacodynamics of IM, IV, and oral administration of TXA in pregnant women

The Woman‐PharmacoTXA is a prospective, randomized, open‐label trial that aims to provide information on the pharmacokinetics, pharmacodynamics, safety, and efficacy of TXA administered by IV, IM, or oral routes in adult women giving birth by cesarean section with at least one risk factor for PPH. Women were randomized to receive one of the following about 1 h prior to cesarean section: 1 g TXA IV, 1 g TXA IM, 4 g TXA oral solution, or no TXA. TXA concentration in maternal blood samples was measured at baseline and at eight time points during 24 h after receipt of intervention. Blood TXA concentration was measured from the umbilical cord to assess placental transfer and heel prick to assess neonate blood levels. The primary endpoint is maternal blood TXA concentrations over time. Secondary outcomes include umbilical cord and neonate TXA concentration, D‐dimer concentration, blood loss, clinical diagnosis of PPH, injection site reactions, and maternal and neonatal adverse events. The trial was conducted in Zambia and Pakistan. Recruitment for this trial is completed and data analysis is ongoing. Results are expected during the first quarter of 2022.^31^

## 4 FUTURE RESEARCH

PKPB modeling and clinical trials in healthy volunteers and trauma patients have highlighted the potential of IM TXA administration. The WOMAN‐PharmacoTXA trial will report soon on the pharmacokinetics and pharmacodynamics of TXA given intramuscularly and as an oral solution. Some safety and efficacy data will be available from this Phase 2 trial; however, recommendations for use in patients may require larger trials with clinically relevant outcomes.

One disadvantage of IM TXA administration is the need to give two 5 ml injections to deliver 1 g TXA (current formulation is 100 mg/mL). Giving two injections instead of one takes more time and is more inconvenient for patients. The availability of galenic forms adapted for a single injection would be an advance for patients, both in terms of ease of use, efficacy, and tolerance of the treatment. If the 1 g TXA dose could be provided in a smaller volume this would facilitate IM injection.^32^

Development of an autoinjector to facilitate administration of IM TXA by first responders or bystanders will allow women with PPH to benefit from IM TXA if shown to be effective. Autoinjectors have been designed for use by anyone in an emergency, including people who are not medically trained, such as a friend, family, bystander, or the person themselves (if they are well enough). Currently, research on autoinjectors for use with TXA in trauma is ongoing and manufacturers are working on the development of large‐volume autoinjectors.^33^, ^34^

## 5 CONCLUSION

In this review we discuss the TXA plasma concentration needed to inhibit fibrinolysis. We showed that plasma TXA concentrations over 10 mg/L provide near maximal inhibition of fibrinolysis, with concentrations over 5 mg/L providing partial inhibition. Oral (tablets and solution) and subcutaneous routes take too long to reach therapeutic levels to be used in the emergency PPH situation. Whereas IM TXA is rapidly and completely absorbed in healthy volunteers and achieved therapeutic concentration within 11 min in shocked, bleeding trauma patients. If absorption was similarly rapid in pregnant women, this would strongly suggest the IM route as an alternative to IV TXA. The Woman‐PharmacoTXA trial, which will report soon, will provide information on the pharmacokinetics and pharmacodynamics of TXA given intramuscularly and as an oral solution in pregnant women. It will also provide information on placenta transfer and elimination by the neonate.

In conclusion, the pharmacokinetic profile of IM TXA is extremely favorable as an alternative to IV administration. Additionally, IM TXA administration is easier than IV because there is no need for cannulation or slow injection (which takes 10 min). It can be performed by nursing staff who are not allowed to give IV drugs.

## CONFLICT OF INTEREST

The authors declare no conflicts of interest.

## AUTHOR CONTRIBUTIONS

HSS and IR conceived the study and agreed the studies to be included in this review. All authors contributed (HSS, SDG, KM, HKA, DF, MA, and IR) to the literature review and drafting the paper.

## Supporting information


Data S1

## References

[1] Say L , Chou D , Gemmill A , et al. Global causes of maternal death: A WHO systematic analysis. Lancet Glob Health. 2014;2(6):323‐333. 10.1016/S2214-109X(14)70227-X 25103301

[2] Geller SE , Koch AR , Garland CE , MacDonald EJ , Storey F , Lawton B . A global view of severe maternal morbidity: moving beyond maternal mortality. Reprod Health. 2018;15(Suppl 1):98. 29945657 10.1186/s12978-018-0527-2 PMC6019990

[3] World Health Organization . The Prevention and Management of Postpartum Haemorrhage. Report of a Technical Working Group. Geneva 3–6 July, 1989. Accessed November 28, 2021. https://apps.who.int/iris/bitstream/handle/10665/61409/WHO_MCH_90.7.pdf

[4] Atuoye KN , Dixon J , Rishworth A , Galaa SZ , Boamah SA , Luginaah I . Can she make it? Transportation barriers to accessing maternal and child health care services in rural Ghana. BMC Health Serv Res. 2015;15(1):333. 26290436 10.1186/s12913-015-1005-y PMC4545969

[5] Mannucci PM . Hemostatic drugs. N Engl J Med. 1998;339(4):245‐253. 9673304 10.1056/NEJM199807233390407

[6] Ker K , Edwards P , Perel P , Shakur H , Roberts I . Effect of tranexamic acid on surgical bleeding: systematic review and cumulative meta‐analysis. BMJ. 2012;344:e3054. 22611164 10.1136/bmj.e3054 PMC3356857

[7] Ker K , Prieto‐Merino D , Roberts I . Systematic review, meta‐analysis and meta‐regression of the effect of tranexamic acid on surgical blood loss. Br J Surg. 2013;100(10):1271‐1279. 23839785 10.1002/bjs.9193

[8] CRASH‐2 trial collaborators , Shakur H , Roberts I , et al. Effects of tranexamic acid on death, vascular occlusive events, and blood transfusion in trauma patients with significant haemorrhage (CRASH‐2): a randomised, placebo‐controlled trial. Lancet. 2010;376:23‐32. 20554319 10.1016/S0140-6736(10)60835-5

[9] CRASH‐2 collaborators , Roberts I , Shakur H , et al. The importance of early treatment with tranexamic acid in bleeding trauma patients: an exploratory analysis of the CRASH‐2 randomised controlled trial. Lancet. 2011;377(9771):1096‐1101. 21439633 10.1016/S0140-6736(11)60278-X

[10] CRASH‐3 trial collaborators . Effects of tranexamic acid on death, disability, vascular occlusive events and other morbidities in patients with acute traumatic brain injury (CRASH‐3): a randomised, placebo‐controlled trial. Lancet. 2019;394;10210:1713–1723. Erratum in: Lancet. 2019;394;10210:1712. 31623894 10.1016/S0140-6736(19)32233-0 PMC6853170

[11] Bryant‐Smith AC , Lethaby A , Farquhar C , Hickey M . Antifibrinolytics for heavy menstrual bleeding. Cochrane Database Syst Rev. 2018;4:CD000249. 29656433 10.1002/14651858.CD000249.pub2 PMC6494516

[12] Taeuber I , Weibel S , Herrmann E , et al. Association of intravenous tranexamic acid with thromboembolic events and mortality: a systematic review, meta‐analysis, and meta‐regression. JAMA Surg. 2021;156(6):e210884. 10.1001/jamasurg.2021.0884 PMC8047805 33851983

[13] Woman Trial Collaborators . Effect of early tranexamic acid administration on mortality, hysterectomy, and other morbidities in women with post‐partum haemorrhage (WOMAN): an international, randomised, double‐blind, placebo‐controlled trial. Lancet. 2017;389(10084):2105‐2116. 28456509 10.1016/S0140-6736(17)30638-4 PMC5446563

[14] Shakur H , Beaumont D , Pavord S , Gayet‐Ageron A , Ker K , Mousa HA . Antifibrinolytic drugs for treating primary postpartum haemorrhage. Cochrane Database Syst Rev. 2018;2:CD012964. 29462500 10.1002/14651858.CD012964 PMC6491317

[15] Nilsson IM . Clinical pharmacology of aminocaproic and tranexamic acids. J Clin Pathol Suppl (R Coll Pathol). 1980;14:41‐47. 7000846 PMC1347104

[16] Ahmadzia HK , Luban NLC , Li S , et al. Optimal use of intravenous tranexamic acid for hemorrhage prevention in pregnant women. Am J Obstet Gynecol. 2021;225:85.e1‐85.e11. 33248975 10.1016/j.ajog.2020.11.035 PMC8149481

[17] World Health Organization . WHO recommendation on tranexamic acid for the treatment of postpartum haemorrhage. WHO; 2017. 29630190

[18] Gayet‐Ageron A , Prieto‐Merino D , Ker K , et al. Effect of treatment delay on the effectiveness and safety of antifibrinolytics in acute severe haemorrhage: a meta‐analysis of individual patient‐level data from 40 138 bleeding patients. Lancet. 2017;391(10116):125‐132. 29126600 10.1016/S0140-6736(17)32455-8 PMC5773762

[19] Picetti R , Shakur‐Still H , Medcalf RL , Standing JF , Roberts I . What concentration of tranexamic acid is needed to inhibit fibrinolysis? A systematic review of pharmacodynamics studies. Blood Coagul Fibrinolysis. 2019;30(1):1‐10. 30585835 10.1097/MBC.0000000000000789 PMC6365258

[20] Kane Z , Picetti R , Wilby A , et al. Physiologically based modelling of tranexamic acid pharmacokinetics following intravenous, intramuscular, sub‐cutaneous and oral administration in healthy volunteers. Eur J Pharm Sci. 2021;1(164):105893. 10.1016/j.ejps.2021.105893 PMC8299544 34087356

[21] Pilbrant A , Schannong M , Vessman J . Pharmacokinetics and bioavailability of tranexamic acid. Eur J Clin Pharmacol. 1981;20(1):65‐72. 7308275 10.1007/BF00554669

[22] Grassin‐Delyle S , Semeraro M , Lamy E , et al. Pharmacokinetics of tranexamic acid after intravenous, intramuscular and oral routes: a prospective, randomized, cross‐over trial in healthy volunteers. Br J Anaesth. 2022 Jan 5 [Epub ahead of print];128:465‐472. 34998508 10.1016/j.bja.2021.10.054

[23] Kazma JM , van den Anker J , Allegaert K , Dallmann A , Ahmadzia HK . Anatomical and physiological alterations of pregnancy. J Pharmacokinet Pharmacodyn. 2020;47(4):271‐285. 32026239 10.1007/s10928-020-09677-1 PMC7416543

[24] Davison JM , Dunlop W . Renal hemodynamics and tubular function normal human pregnancy. Kidney Int. 1980;18(2):152‐161. 7003196 10.1038/ki.1980.124

[25] Abduljalil K , Furness P , Johnson TN , Rostami‐Hodjegan A , Soltani H . Anatomical, physiological and metabolic changes with gestational age during normal pregnancy: a database for parameters required in physiologically based pharmacokinetic modelling. Clin Pharmacokinet. 2012;51(6):365‐396. 22515555 10.2165/11597440-000000000-00000

[26] Wishart DS , Feunang YD , Guo AC , et al. DrugBank 5.0: a major update to the DrugBank database for 2018. Nucleic Acids Res. 2018;46(D1):D1074‐D1082. 29126136 10.1093/nar/gkx1037 PMC5753335

[27] Lechien A , Faraoni D , Van der Linden P . Effective tranexamic acid concentration for 95% inhibition of tissue‐type plasminogen activator‐induced hyperfibrinolysis in full‐term pregnant women: a prospective interventional study. Blood Coagul Fibrinolysis. 2021;32(3):186‐193. 33470644 10.1097/MBC.0000000000001015

[28] Muhunthan K , Balakumar S , Navaratnaraja TS , Premakrishna S , Arulkumaran S . Plasma concentrations of tranexamic acid in postpartum women after oral administration. Obstet Gynecol. 2020;135(4):945‐948. 32168220 10.1097/AOG.0000000000003750 PMC7098443

[29] Haljamae H . Microcirculation and hemorrhagic shock. Am J Emerg Med. 1984;2:100‐107. 6517978 10.1016/0735-6757(84)90117-7

[30] Grassin‐Delyle S , Shakur‐Still H , Picetti R , et al. Pharmacokinetics of intramuscular tranexamic acid in bleeding trauma patients: a clinical trial. Br J Anaesth. 2021;126(1):201‐209. 33010927 10.1016/j.bja.2020.07.058

[31] Arribas M , Roberts I , Chaudhri R , et al. WOMAN‐PharmacoTXA trial: Study protocol for a randomised controlled trial to assess the pharmacokinetics and pharmacodynamics of intramuscular, intravenous and oral administration of tranexamic acid in women giving birth by caesarean section. Wellcome Open Res. 2021;16(6):157. 10.12688/wellcomeopenres.16884.1 PMC8264807 34250266

[32] National Center for Biotechnology Information . PubChem. Compound Summary. Tranexamic acid. Accessed November 28, 2021. https://pubchem.ncbi.nlm.nih.gov/compound/Tranexamic‐acid.

[33] Army Technology [website] . New TXA Autoinjector technology to stop blood loss on battlefield. April 18, 2019. Accessed November 28, 2021. https://www.army‐technology.com/news/txa‐autoinjector‐blood‐loss/

[34] MACH32 Autoinjectors [website]. Accessed November 28, 2021. https://www.mach32.net/autoinjectors

