# Supplementary material for: Alternative routes to intravenous tranexamic acid for postpartum hemorrhage: A systematic search and narrative review
Source: Int J Gynaecol Obstet. 2022 Jun 28;158(Suppl 1):40–5. doi: 10.1002/ijgo.14201 (PMC9327714; doi:10.1002/ijgo.14201)
Supplement: Supplementary file 1 — Data S1 [file IJGO-158-40-s001.docx]

**Supporting information S1: Search Strategy**

**Ovid MEDLINE(R) ALL**

1. Tranexamic Acid/pk 59
2. Tranexamic Acid/ad, pd and *Fibrinolysis/de 91 3 1 or 2 147
3. Tranexamic Acid/ 4294
4. (Tranexamic or TXA or AMCA or AMCHA or cyklokapron).ti,ab,kf,rn. 7457

6 4 or 5 7457

1. (fibrinolysis or fibrinolytic or hyperfibrin* or hypofibrin*).ti,ab,kf,hw. 70522
2. (fibrin* adj3 (activat* or activity or bind* or challenge or degrad* or inhibit* or reduc*)).ti,ab,kf. 24767
3. (CLT or (clot adj (formation or breakdown or degrad* or inhibit* or lysis or reduc*))).ti,ab,kf. 7539
4. Fibrin Fibrinogen Degradation Products/9731
5. (FDP or (fibrin* adj3 degrad*) or D-Dimer*).ti,ab,kf. 20521
6. (coagulation potential or coagulability).ti,ab,kf.1342
7. 7 or 8 or 9 or 10 or 11 or 12 101085
8. blood coagulation tests/ 20086
9. (((blood or plasma or laboratory or point-of-care) adj2 test) or assay or immunoassay or ELISA).ti,ab,kf. 944825
10. exp Immunoassay/ 496674
11. Thrombelastography/ 5763
12. (thromboelastogra* or TEG or thromboelastomet* or ROTEM or halo assay).ti,ab,kf. 6390
13. (h?emostasis potential or OHP or turbidity or global fibrinolytic capacity or GFC).ti,ab,kf. 14167
14. (fibrin* adj3 (in-vivo or in-vitro or ex-vivo)).ti,ab,kf. 1757

21 14 or 15 or 16 or 17 or 18 or 19 or 20 1321695

1. (Tranexamic or TXA).mp. and concentration*.ti,ab,kf. 630
2. ((blood or plasma or exogenous) adj5 (concentration? or availability)).ti,ab,kf. 253659
3. Tranexamic Acid/an, bl [Analysis, Blood] 71
4. Dose-Response Relationship, Drug/ 417191
5. (dose? or megadose? or dosing).ti,ab,kf.1453713

27 (pharmacokinetic? or pharmacodynamic? or drug kinetic? or bioavailability).ti,ab,kf,hw. 251332

28 22 or 23 or 24 or 25 or 26 or 27 1987490

29 6 and 13 and (21 or 28) 573

1. (administ* or inject? or route or intraveneous or oral* or ingestion or sublingual or intraosseous or intramuscular or subcutaneous or transdermal).ti,ab,kf. 2307261
2. (healthy or volunteer? or phase 1 study).ti,ab,kf. 995844

32 6 and 30 and 3171

1. (screen* or detect*).ti,ab,kf,hw.3304712
2. (precise or precision or accurate or accuracy or reproducibility or reproducible or sensitiv* or specific* or validat* or validity or reliable or reliability or standard*).ti,ab,kf. 6870634
3. "Reproducibility of Results"/ 434693
4. exp "Sensitivity and Specificity"/ 627339

37 33 or 34 or 35 or 36 9026570

38 29 and 37 162

39 3 or 32 or 38 346

1. (exp Animals/ or Animal Experimentation/ or exp Models, Animal/) not (exp Human Experimentation/ or Humans/) 4941309
2. ((rat or rats or rodent* or mouse or mice or murine or rabbit or rabbits or pigs or piglets or swine or porcine or sheep or lambs or ovine or cat or cats or feline or dog or dogs or canine or horse? or equine or cattle or bovine or monkey or marmoset?) not (human? or man)).ti. 2114132

42 39 not (40 or 41) 291

43 limit 42 to ed=20171106-20210731 91

# **Embase**

1. ((Tranexamic or TXA) adj3 concentration?).ti,ab,kw. 166
2. tranexamic acid/cr, do, pk, pd [Drug Concentration, Drug Dose, Pharmacokinetics, Pharmacology] 1386
3. *fibrinolysis/ or fibrinolysis.ti. 16979

4 (1 or 2) and 3 104

1. tranexamic acid/ 15675
2. (Tranexamic or TXA or AMCA or AMCHA or cyklokapron).ti,ab,kw,rn. 16901

7 5 or 6 16945

1. (fibrinolysis or fibrinolytic or hyperfibrin* or hypofibrin*).ti,ab,kw,hw. 93477
2. (fibrin* adj3 (activat* or activity or bind* or challenge or degrad* or inhibit* or reduc*)).ti,ab,kw. 29988

10 FDP.ab. 3934

1. fibrin degradation product/ 4398
2. D dimer/ 30781
3. d-dimer?.ti,ab,kw. 23940
4. (coagulation potential or coagulability).ti,ab,kw,hw. 1547

15 8 or 9 or 10 or 11 or 12 or 13 or 14 139492

1. blood examination/ 15993
2. blood clotting test/ 11211
3. ((blood or plasma or laboratory) adj2 test?).ti,ab,kw. 116457
4. laboratory test/ or exp immunoassay/ or enzyme linked immunosorbent assay/ 812946
5. "point of care testing"/ 16961
6. (assay or immunoassay or ELISA).ti,ab,kw,hw. 1708015
7. thromboelastography/ 8857
8. (thromboelastogra* or TEG or thromboelastomet* or ROTEM or halo assay).ti,ab,kw. 10360
9. (h?emostasis potential or OHP or turbidity or global fibrinolytic capacity or GFC).ti,ab,kw. 17693
10. (in-vivo or in-vitro or ex-vivo).ti,ab,kw,hw. 2931794

26 16 or 17 or 18 or 19 or 20 or 21 or 22 or 23 or 24 or 25 4568075

1. concentration response/ or dose response/ or *drug response/ 546609
2. exp *pharmacokinetic parameters/ 64838
3. pharmacokinetic parameters/ or maximum plasma concentration/ or minimum plasma concentration/ or time to maximum plasma concentration/ 60638
4. drug blood level/ or drug concentration/ 227774
5. dosage schedule comparison/ or drug dose/ 33324
6. (dose or megadose or dosing).ti,ab,kw,hw. 2319639
7. exp pharmacokinetics/ 793672
8. exp pharmacodynamics/ 3823934
9. (pharmacokinetic? or pharmacodynamic? or drug kinetic? or bioavailability).ti,ab,kw. 342769
10. ((blood or plasma or exogenous) adj5 (concentration? or availability)).ti,ab,kw. 310383
11. (concentration? or bioavailability).ti. 201244

38 27 or 28 or 29 or 30 or 31 or 32 or 33 or 34 or 35 or 36 or 37 5928375

39 7 and 15 and 26 and 38 387

1. diagnostic accuracy/ 275402
2. "sensitivity and specificity"/ 416871
3. exp reliability/ 203301
4. (screen* or detect*).ti,ab,kw,hw. 4536082
5. (precise or precision or accurate or accuracy or reproducibility or reproducible or sensitiv* or specific* or validat* or validity or reliable or reliability or standard*).ti,ab,kw. 8740866
6. Gold standard/ 61627
7. consensus/ 82744
8. validity/ 65778
9. screening/ or screening test/ 255010
10. intermethod comparison/ 278473
11. practice guideline/ 481823

51 40 or 41 or 42 or 43 or 44 or 45 or 46 or 47 or 48 or 49 or 50 11980337

52 39 and 51 156

1. phase 1 clinical trial/ 62103
2. normal human/ 752960
3. (healthy or volunteer?).ti,ab,kw. 1372116

56 53 or 54 or 55 1934429

57 ((Tranexamic or TXA or AMCA or AMCHA or cyklokapron) adj3 (administ* or inject? or route or intraveneous or oral* or ingestion or sublingual or intraosseous or intramuscular or subcutaneous or transdermal)).ti,ab,kw.1851

58 56 and 57 63

1. Animal experiment/ not (human experiment/ or human/) 2378629
2. ((rat or rats or rodent* or mouse or mice or murine or rabbit or rabbits or pigs or piglets or swine or porcine or sheep or lambs or ovine or cat or cats or feline or dog or dogs or canine or horse? or equine or cattle or bovine or monkey or marmoset?) not (human? or man)).ti. 2228410

61 59 or 60 3522502

62 (4 or 52 or 58) not 61 264

63 limit 62 to em=201745-202130 83

# **Web of Science**

*Applied limits to 17 for publication added between 6 November 2017 to 31 July 2021.*

17 #9 OR #13 OR #16 105

16 #14 AND #15 21

15 TS=((concentration* or bioavailability or pharmacokinetic* or pharmacodynamic*)) 3,417,741

14 TS=(Tranexamic SAME (healthy and (volunteer* or subject*))) 56

13 #12 AND #11 AND #10 3

12 TI=(administ* or inject* or route or intravenous or oral* or ingestion or

sublingual or intraosseous or intramuscular or subcutaneous or transdermal or dose or megadose or dosing) 1,144,405

11 ALL=("healthy volunteer*") 87,280

10 TI=(Tranexamic or TXA or AMCA or AMCHA or cyklokapron) 3,955

9 #1 AND #5 AND #6 AND #7 AND #8

8 TS=(diagnos* or screen* or detect* or precise or precision or accurate or accuracy or reproducibility or reproducible or sensitiv* or specific* or validat* or validity or reliable or reliability or standard* or consensus or comparison) 18,690,087

7 TS=(“concentration response” or “dose response” or “drug response” or “plasma

concentration” or “blood level” or “drug concentration” or “dosage schedule” or dose or megadose or dosing or pharmacokinetic* or pharmacodynamic* or “drug kinetic*” or bioavailability) or TS=((blood or plasma or exogenous) same (concentration? or availability)) OR TI=(concentration*) 2,228,916

6 TS=(((blood or plasma or laboratory) same test* ) or (assay or immunoassay or ELISA or thromboelastogra* or TEG or thromboelastomet* or ROTEM or “halo assay” or “hemostasis potential” or “haemostasis potential” or OHP or turbidity or “global fibrinolytic capacity” or GFC or in-vivo or in-vitro or ex-vivo)) 4,017,560

5 #2 OR #3 OR #4 91,569

4 TS=((FDP or d-dimer* or “coagulation potential” or coagulability)) 19,336

3 TS=(fibrin* same (activat* or activity or bind* or challenge or degrad* or inhibit* or reduc*)) 66,177

2 TS=(fibrinolysis or fibrinolytic or hyperfibrin* or hypofibrin*) 37,269

1 TS=(Tranexamic or TXA or AMCA or AMCHA or cyklokapron) 9,950
